# Retraction Note: Effect of catalpol on remyelination through experimental autoimmune encephalomyelitis acting to promote Olig1 and Olig2 expressions in mice

**DOI:** 10.1186/s12906-023-04196-1

**Published:** 2023-10-07

**Authors:** Tao Yang, Qi Zheng, Su Wang, Ling Fang, Lei Liu, Hui Zhao, Lei Wang, Yongping Fan

**Affiliations:** 1 Department of Traditional Chinese Medicine, Beijing Tiantan Hospital, Capital Medical University, Beijing, 100050 People’s Republic of China; 2 School of Traditional Chinese Medicine, Beijing Key Lab of TCM Collateral Disease Theory Research, Capital Medical University, Beijing, 100069 People’s Republic of China; 3 Oncology Department, Guang An Men Hospital of China Academy of Chinese Medical Sciences, Beijing, 100053 People’s Republic of China

**Retraction Note: BMC Complement Med Ther 17, 240 (2017).**

10.1186/s12906-017-1642-2

The Editor has retracted this article. After publication, concerns were raised about three of the figures:

- Panels A1 and F1 in Fig. 8 appear to be duplicates.
- Panels D and F in Fig. 6 appear to overlap.
- Panel E2 in Fig. 2 appears to be identical with Fig. 3 in [1].

The authors have provided data files. However, these have not allayed the concerns and the Editor therefore no longer has confidence in the results and conclusions presented. None of the authors has responded to correspondence from the Editor about this retraction.
